# A multicriteria decision analysis (MCDA) tool to purchase implantable medical devices in Egypt

**DOI:** 10.1186/s12911-022-02025-y

**Published:** 2022-11-09

**Authors:** Baher Elezbawy, Ahmad Nader Fasseeh, Bertalan Németh, Mary Gamal, Mariam Eldebeiky, Remonda Refaat, Amr Taha, Shimaa Rabiea, Marwa Abdallah, Soha Ramadan, Hasnaa Noaman, Amany Bahaa Eldin, Hossam Mostafa, Sara Nouh, Asmaa Zaki, Mohamed Abdelrahman, Sherif Abaza, Zoltàn Kalò

**Affiliations:** 1 Syreon Middle East, Alexandria, Egypt; 2 Faculty of Social Sciences, Eötvös Loránd University, Budapest, Hungary; 3 Syreon Research Institute, Budapest, Hungary; 4 The Egyptian Authority for Unified Procurement, Medical Supply, and Technology Management, Cairo, Egypt; 5 Ministry of Health and Population, Cairo, Egypt; 6 Suez Canal University Hospital, Ismailia, Egypt; 7 Zagazig University, Zagazig, Egypt; 8 Egyptian Drug Authority, Cairo, Egypt; 9 Health Insurance Organization, Cairo, Egypt; 10 The General Authority of Health care, Cairo, Egypt; 11 Syreon Middle East, Cairo, Egypt; 12 Center for Health Technology Assessment, Semmelweis University, Budapest, Hungary

**Keywords:** MCDA, Multicriteria decision analysis, Medical devices, Implantable, Procurement

## Abstract

**Background:**

With the availability of several similar medical devices performing the same function, choosing one for reimbursement is not easy, especially if purchased for a large number of patients. The objective of this project was to create a multicriteria decision analysis (MCDA) tool, that captures and compares all implantable medical devices’ attributes, to provide an objective method for choosing among the available options in Egypt.

**Method:**

We conducted a systematic review and expert interviews, to identify the relevant criteria for inclusion in the tool. Subsequently, a workshop was conducted, that involved experts in procuring and tendering medical devices. Experts chose the criteria, ranked them, assigned weights and scoring functions for each criterion, and then created the draft tool. A pilot phase followed; then, another workshop was conducted to fine-tune the tool. We readjusted the tool based on experts’ experience with the draft tool.

**Results:**

The final tool included eight criteria, arranged according to their weightage: technical characteristics (29.4%), country of origin (19.5%), use in reference countries (14.9%), supply reliability (11.7%), previous use in tenders (9.0%), instant replacement within product variety (6.9%), pharmacovigilance (4.6%), and refund or replacement (4.0%). Each medical device was assessed on these eight criteria to achieve a final score, that was compared to the alternative devices’ scores. Price is not included in the MCDA tool, but it will be added in the financial evaluation phase.

**Conclusion:**

Decisionmakers could use the MCDA tool, to make evidence-based and objective decisions for purchasing implantable devices, in the Egyptian public sector. Post price evaluation, the product with the best value will be chosen for reimbursement.

**Highlights:**

- We created an MCDA tool to help decision makers choose between alternative implantable medical devices in Egypt.
- The MCDA tool includes eight criteria, where price is evaluated as a separate step.
- “Technical characteristics” and “country of origin” criteria carried the highest weights, thus representing approximately 50% of the decision.

**Supplementary Information:**

The online version contains supplementary material available at 10.1186/s12911-022-02025-y.

## Introduction

Thousands of medical devices currently help healthcare professionals achieve improved outcomes. Medical devices range from simple low-risk devices (e.g., bandages and wheelchairs) to complex high-risk devices (e.g., pacemakers and coronary stents) [1]. According to the World Health Organization, a medical device is an instrument, machine, apparatus, or implant, intended for medical purposes [2]. An implantable medical device, such as a cardiac defibrillator or implantable insulin pump, is defined as a device that is introduced inside the body [3].

There is widespread availability of equivalent medical devices in a broad price range, specifications, and qualities [4]. In a world with scarce resources, reimbursing all novel yet expensive medical devices is not always feasible. Healthcare professionals purchasing medical devices, need an objective tool to compare available alternatives and choose the best option [5].

Multicriteria decision analysis (MCDA excellently solves this dilemma, especially in the evaluation of expensive, high-risk medical devices [6]. Currently, MCDA tools are being increasingly used in the healthcare sector [7]. They have been successfully used in purchasing off-patent pharmaceuticals [8], medical devices [6], and orphan drugs [9].

An MCDA tool is created by choosing a list of criteria relevant to the decision problem. The criteria are then ranked and weighted according to their relative importance. For each criterion, a predefined scoring function is set, where each assessed option is eligible to receive a score based on a precise rule (i.e., a rule to define which score each compared item receives) [10].

The healthcare system in Egypt is rapidly reforming, perceived in the kickoff of the Universal Health Insurance (UHI), Egyptian Drug Authority (EDA), and Egyptian Authority for Unified Procurement, Medical Supply, and Technology Management (UPA) [11, 12]. UPA will have high negotiation power, with its responsibility to procure all medical supplies for Egypt’s governmental sector, including medical devices [12].

Owing to the country’s population of over 100 million, a great demand for treatment and an increase in the market size of the healthcare sector are expected [11]. Egypt has a high demand for cost-effective and efficient medical devices. This can be achieved by developing an MCDA tool similar to the previously implemented one used for purchasing out-of-patent oncology medicines in Egypt [13].

Several examples of MCDA tools or similar concepts are used in purchasing medical devices worldwide [14–16], yet no tools exist for purchasing medical devices in Egypt. Tools of product value assessment are not easily transferrable between different jurisdictions [17]. Similar MCDA tools developed in other countries, cannot be transferred and used in Egypt; however, they can guide to create a new tool that reflects local requirements, and corresponds to the country-specific decision problem.

This study aims to create an objective and user-friendly MCDA tool, to help decision makers in the public purchase of Egypt’s implantable medical devices.

## Methods

This MCDA tool was created through the participation of Egyptian healthcare decision-makers, in two workshops conducted in 2021 and 2022.

**Fig. 1:**
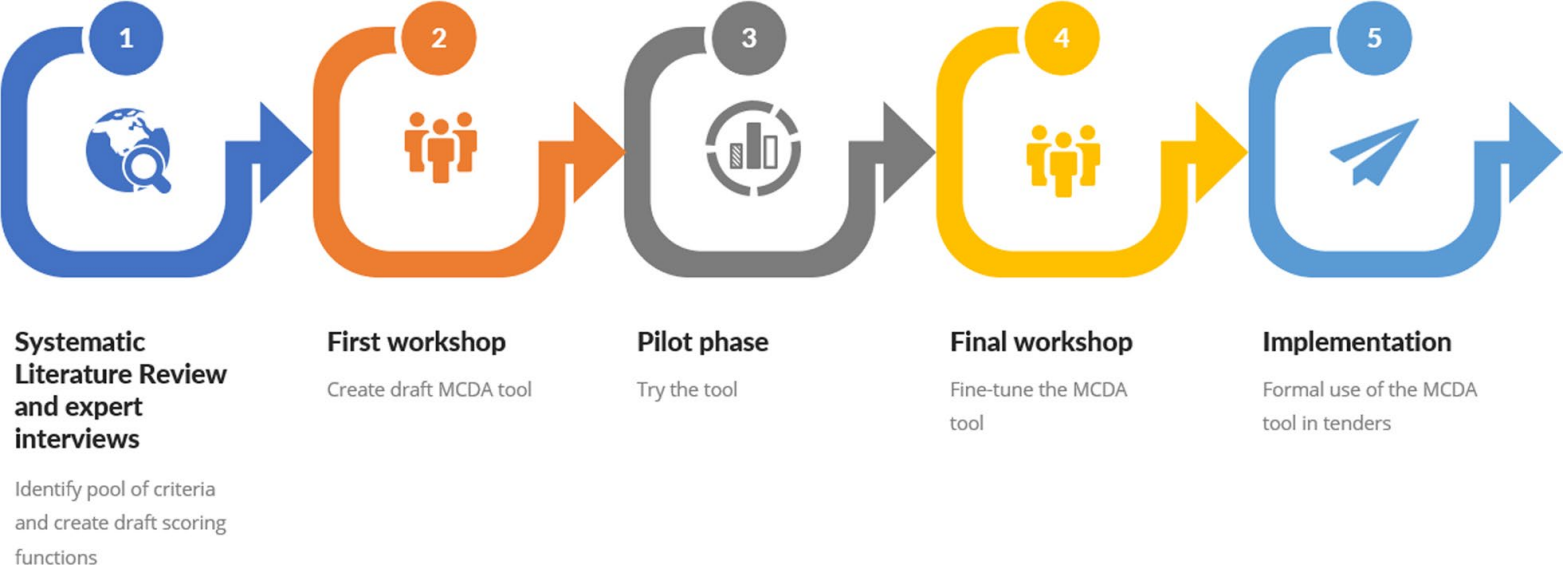
Project phases The project phases were: (1) Systematic review and expert interviews, (2) First workshop, (3) Pilot phase, (4) Final workshop, and (5) Implementation

First, a systematic literature review and three online expert interviews were conducted, to define the pool of criteria used as initiation for the tool’s creation in the workshops. We then conducted a face-to-face preparatory workshop with experts, to create a draft MCDA tool. Users tried the tool through a pilot period, followed by a second face-to-face workshop held to readjust the tool, and create a final version for formal usage in tenders. The project phases are illustrated in Fig. 1.

### Research phase (systematic literature review and expert interviews)

To prepare for the workshops, research was conducted to create a pool of relevant criteria with proposed scoring functions. We conducted a systematic review to identify all studies and reports discussing the use of the MCDA approach in purchasing and/or prioritizing medical devices. We extracted the relevant criteria and their scoring functions from eligible references.

We searched the Medline and Embase databases using PubMed and Scopus search engines. Additionally, to identify any potential missing literature, the first 100 hits from the Google Scholar search engine were identified and screened. The search was conducted on the 30^th^ of September 2020. We restricted the search to English-language publications but not by time or geographical location. Detailed search terms are provided in Additional file 1: Appendix 1.

The titles and abstracts of the identified studies were screened, and eligible studies were included in the full-text screening and data extraction phases. The exclusion criteria were as follows: (1) studies with no English abstract, (2) studies not discussing MCDA or decision criteria, (3) studies not involving MCDA tools for medical devices, and (4) studies not discussing purchasing or prioritization of medical devices. Two independent reviewers screened the title and abstract of each study, and the third principal researcher consolidated the conflicts.

During the full-text screening and data extraction phases, each study was screened and extracted by one researcher and revised for completeness and accuracy by another independent researcher. The same exclusion criteria used for the title and abstract screening phase were used for full-text screening, in addition to excluding inaccessible studies. We checked the references of the included studies for eligibility to ensure that there were no missing potential studies (snowball searching).

After data extraction, we categorized and grouped the identified criteria into domains and subdomains for ease of analysis. The systematic review findings were used as a guide to create a primary list of criteria for comparing alternative devices in Egypt.

Subsequently, three online expert interviews were conducted to formulate a preliminary list of criteria based on local settings, previous experience with scoring tools, and guidance from the systematic review. The experts interviewed were highly experienced (with more than ten years of experience) and up-to-date with medical device tendering and registration processes in Egypt.

### Creating the MCDA tool

We created an MCDA tool to provide results for the scores of several compared medical devices and advise on the best option to procure. The tool was created using Microsoft Excel. This tool requires the following inputs: (1) criteria names, (2) criteria ranking according to importance, (3) the weight of each criterion, (4) how each criterion is scored (scoring functions), and (5) scores for each scoring function.

The MCDA tool uses these inputs to create a summary table for each comparator. Each comparator is assessed and provides a score for each criterion (for example, if we are assessing an implantable device for the “supply reliability” criterion, and the weight of this criterion is 10%, the assessed device will receive a score out of this 10% based on its performance. If the device has no problems in supply, it will receive 10%, whereas if it has problems in supply, it should receive 1%, 5%, 6%, or 8% of this criterion’s score based on the frequency of supply problems. These values (1%-10%) depend on the predefined scoring function.)

Finally, each device should be similarly assessed for all criteria, and the score is aggregated to provide a final score out of 100%. This score can be compared with the scores of other available medical devices. A summary table and graphical representation are automatically generated to provide the final results.

To create the MCDA tool, inputs of criteria, ranks, weights, and scoring functions are required. We conducted workshops with experts to define these inputs and to add them to the tool.

### First workshop

We conducted a two-day workshop with 20 public healthcare and medical device tendering experts on the 23rd of January and 6th of February 2021.

Workshop participants discussed the systematic review findings during the workshop to understand how medical devices were being assessed or prioritized in different jurisdictions. Subsequently, participants were introduced to the preliminary proposed list of criteria. Participants were allowed to vote for potential modifications to the list and for the inclusion or exclusion of specific criteria. Voting was conducted anonymously, using the Mentimeter® online platform. Participants’ request for modifications was reviewed through votes, and if more than half of the participants agreed, the modification was adopted.

After criteria selection, experts voted to rank them. The proposed list of criteria needed to be ranked according to their importance. After voting as per the descending order of importance, the average was calculated and presented. The participants then voted for each criterion’s scoring function and weight.

For scoring functions, each device is assigned a score from 0 to 100% of the criterion’s weight depending on the device’s achieved outcomes(i.e., for example, if there are three options for scoring a criterion: least achiever, medium achiever, and highest achiever, the scores may be voted as 0%,50% and 100%, respectively; thus, when a device is assessed, it may receive either 0%, 50%, or 100% of the criterion’s weight based on its specifications). Some scoring functions were set as exclusion criteria (i.e., if the medical device scored this item, it was excluded from the tender, regardless of its score in any other criteria).

Criteria were weighted relative to each other using the SMART (Simple Multi-Attributable Rating Technique) and swing method [18–20]. Participants voted for the importance of each criterion, compared to the following ranked criterion on a scale of 0% to 100%, where 0% portrays the equal importance of the two compared criteria, and 100% indicates the higher ranked criterion to have double the importance of the lower criterion. Finally, a draft MCDA tool was created based on experts’ understanding and votes.

### Pilot phase

The experts were provided with the Microsoft Excel MCDA tool, developed based on their ranking and votes, to start testing it in a pilot phase. Experts were asked to test the tool on real products, check its results, and assess how the results differed from the results of the previous methodology of choice, which mainly depended on expert opinions. Based on these differences, different weights and scoring functions were tested to observe the potential impact of changing such values on the final score. After trying the tool in several cases, experts suggested a set of possible modifications to the tool. These fine-tunes were set to be discussed in the final workshop in 2022 to formulate a final tool that matches the healthcare system’s exact needs.

### Final workshop

The final two-day workshop was conducted on the 1st and 2nd of March 2022, involving 14 experts. The previously conducted MCDA tool was used as the starting point for the workshop. Experts started modifying the tool and adjusting the criteria, ranks, weights, and scoring functions to match what they expected from the tool.

During the pilot phase, experts provided comments and required modifications to the tool. These suggestions were discussed during the workshop, and voting on the Mentimeter® online platform was initiated for points, where participants lacked consensus. The tool was readjusted based on anonymous votes, and a final tool was created to reflect stakeholder preferences.

## Results

### Systematic literature review and expert interviews

**Fig. 2:**
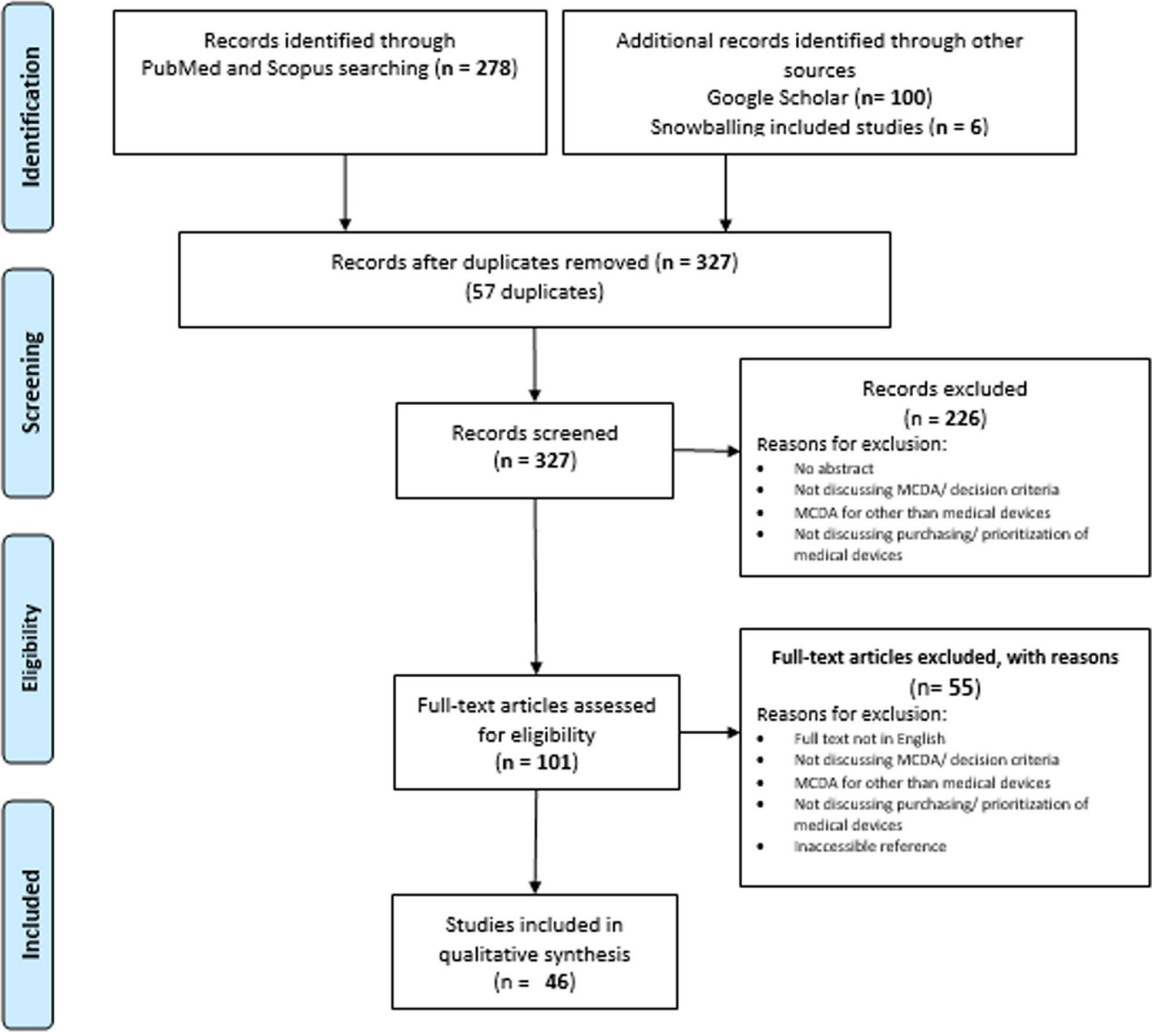
Screening process We screened 278 references identified from Scopus and PubMed, in addition to the first 100 hits from Google Scholar. Six studies were identified through snowball searching. After deduplication, 327 references were screened through titles and abstracts. Of those, 101 were eligible for full text screening. Finally, 46 were included in the data analysis phase

The systematic literature review identified 384 studies, 278 of which were identified from Medline and Embase, 100 from Google Scholar, and six from snowball searching. Of these, only 46 were considered relevant and eligible for data extraction and analysis. The details of the screening process are shown in Fig. 2.

The identified criteria were analyzed and categorized into nine domains (eight main domains and an “others” domain for criteria not fitting in any of the eight main domains). The criteria were categorized to avoid redundancy and facilitate the analysis. Each domain included several criteria that were grouped together.

**Fig. 3:**
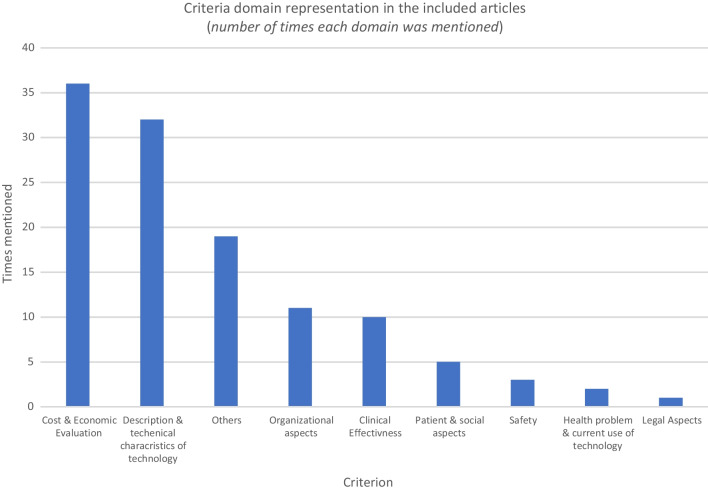
Criteria domain representation in the studies included in the systematic review. “Cost and economic evaluation” was the most common criterion in the tools identified, followed by technical characteristics, organizational aspects, clinical effectiveness, patients and social aspects, safety, health problem and current use of technology, and legal aspects

The cost of the medical device was the most common domain identified in the MCDA tools, followed by the technical characteristics of the medical device. Figure 3 shows the frequencies of each domain in the included studies.

Based on the systematic review findings, criteria from each domain were selected to create the MCDA tool. The criteria included “cost”, “technical characteristics”, “production quality”, “supplier reliability”, “training”, and “refunds”. However, after the expert interviews, “country of origin”, “previous use in reference countries”, “previous use in tenders”, and “instant replacement” criteria were additionally included in the MCDA tool based on local experience in purchasing medical devices.

One significant finding was that in Egypt, the pricing committee is independent of the technical committee, so “cost” criterion could not be included in the MCDA tool with the others. The available alternatives should be assessed for their costs in a separate phase (financial evaluation phase).

Finally, nine criteria were proposed to start the workshop: “technical characteristics of the medical device”, “production quality”, “supply reliability”, “training, refund or replacement”, “country of origin”, “previous use in reference countries”, “previous use in tenders”, and “instant replacement within the product variety”.

### First workshop

The first workshop began with the proposed list of nine criteria. Twenty experts attended, representing the different organizations related to the procuring and tendering of medical devices (UPA, EDA and Ministry of Health and Population (MoHP)). The participants agreed to the inclusion of the “pharmacovigilance” criterion and the exclusion of the “production quality” criterion. “Production quality” criterion was excluded as it was already covered in “use in reference countries” criterion, which is assessed by certificates.

The MCDA tool created upon the participants’ voting included nine criteria as follows: “Technical characteristics of the medical device” (33% of the weight), “Use in reference countries” (17.8%), “Supply reliability” (11.3%), “Country of origin” (9.0%), “Previous use in tenders” (for the product) (7.4%), “Pharmacovigilance system” (7.3%), “Instant replacement within product variety” (within surgery OR on shelf stock) (5.1%), “Training” (4.7%) and “Refund/ replacement” (4.2%).

### Final workshop

Fourteen experts participated in the final workshop. These represent Egypt’s different stakeholders in medical device tendering. They required a few changes compared to the previous workshop’s tool based on the pilot phase, where they used the MCDA tool and checked its validity and applicability in tenders. Participants started discussing the criteria for choosing what should be removed, added, or modified. Their discussions ended up removing one of the proposed criteria, “training,” to reach eight final criteria (Table 1). The “training” criterion was removed from the list because experts agreed that it should be mandatory for all medical device companies to provide training if their devices require that. If training is not provided, the device should be excluded before the assessment using the MCDA tool. Participants also agreed that to ensure training is provided, suppliers should receive part of their payment only after they present a signed document from the healthcare provider (e.g., hospital) that received their device, ensuring that the supplier provided sufficient training for its device.

**Table 1:** Final criteria and scoring functions

Criterion	Scoring options	Score
*Technical characteristics of the medical device* (To assure fulfilling the technical specifications)	Fulfills 100% of the technical specifications required	100%
	Fulfills 90– < 100% of the technical specifications required	80%
	Fulfills 80– < 90% of the technical specifications required	60%
	Fulfills 70– < 80% of the technical specifications required	10%
	Fulfills < 70% of the technical specifications required	Exclusion
*Country of origin*	Reference countries* for both legal and actual manufacturer or local product	100%
	Reference country* of the legal manufacturer or actual manufacturer	75%
	Non reference countries for both	40%
*Use in reference countries* (To assure previous use in countries with good standards of quality)	CFG certificate from FDA	100%
	Canadian free sale certificate + ((medical device active license + MDSAP certificate) or medical device establishment license)	80%
	European CE certificate + free sale certificate from a reference country*	75%
	European CE certificate only (for local products only)	50%
*Supplier reliability* (To assure the reliability of the supplier concerning quantities and delay)	Supplier fulfilled more than 90% of the committed requirements in the last 3 years	100%
	Supplier fulfilled 70–90% of the committed requirements in the last 3 years	80%
	Supplier fulfilled 50–70% of the committed requirements in the last 3 years	60%
	Did not supply previously	50%
	Supplier fulfilled < 50% of the committed requirements in the last 3 years	10%
*Previous use of the product*	Listed in the UPA platform	100%
	Supplied to governmental or non-governmental organizations in the previous 2 years	70%
	Was not supplied previously	45%
*Instant replacement within product variety* (To assure supplier flexibility)	Supplier provides instant replacement within product variety (During surgery on shelf stock)	100%
	Supplier does not provide product replacement for different sizes/ types	15%
*Pharmacovigilance system*		
(EDA will provide evidence for the efficiency of the pharmacovigilance system from 1 year as a maximum)	Supplier has an efficient pharmacovigilance system	100%
	Supplier has a moderate quality pharmacovigilance system	70%
	Supplier has a low-quality pharmacovigilance system	20%
	No pharmacovigilance system	Exclusion
*Refund/Replacement within product variety* (To assure replacing unwanted or expired products)	The product was present in the stagnant report 1 time or less in the last year	100%
	The product was present in the stagnant report 2 times subsequently in the last year	70%
	The product was present in the stagnant report 3 times subsequently in the last year	50%
	The product was present in the stagnant report 4 times in the last year	20%

CFG: Certificate to Foreign Government, FDA: Food and Drug Administration, UPA: The Egyptian Authority for Unified Procurement, Medical Supply, and Technology Management, EDA: Egyptian Drug Authority, CE: Conformitè Europëenne, MDSAP: Medical Device Single Audit Program

*List of reference countries: Australia, Austria, Belgium, Canada, Denmark, Germany, Finland, Iceland, France, Ireland, Luxemburg, The Netherlands, New Zealand, Norway, Sweden, Switzerland, USA, UK, Japan, Italy, Spain, Portugal[21]

The ranking and weightage of different criteria, were also readjusted from the first developed MCDA tool, based on expert discussions.

### Ranking

The following is the final list of criteria:

1. Technical characteristics of the medical device
2. Country of origin
3. Use in reference countries
4. Supply reliability
5. Previous use in tenders
6. Instant replacement within product variety (within surgery OR on shelf stock)
7. Pharmacovigilance system
8. Refund/ replacement

### Scoring

The results of voting on scores that each product can achieve regarding the eight criteria are shown in Table 1. Participants agreed to exclude products fulfilling less than 70% of the required technical specifications from the tender. In addition, products from a supplier that does not have a pharmacovigilance system will be excluded.

### Criteria details and scoring functions

#### Technical characteristics of the medical device

The technical characteristics of implantable devices are crucial to procurement decisions. This was evident from the experts’ ranking and weighting votes for this criterion. Yet “implantable devices” is an umbrella term for a wide range of product classes that are technically incomparable. It would not be feasible to develop a dedicated tool for each medical-device class because of the large number of different product classes available. Therefore, we used an alternative approach to assess the technical characteristics for the MCDA. A dedicated scoring tool for each product class was developed by the technical committee and was used to assess the scores of each product. The percentage of devices fulfilling the technical specifications was calculated and compared for the available devices. (For example, if the procurement agency is purchasing pacemakers, the technical committee lists 20 specifications for the required pacemaker, such as weight (less than 40 g), battery type (lithium), battery longevity (more than 7 years), and size (less than 11 cc) … etc. If the assessed pacemaker achieves only 16 of the required 20 specifications, it fulfils 80% of the required technical specifications).

#### Country of origin

To ensure manufacturing quality, the country of origin of the medical device was assessed. If the medical device comes from a country with high-quality standards, it will receive a high score for this criterion. A list of countries with high-quality standards has been published by the Ministry of Health and Population and is known as the list of reference countries [21].

#### Use in reference countries

For the use in reference countries criterion, to assure the previous use of the medical device in countries with good standards of quality, certifications are required from the medical device’s manufacturer to prove its good quality. Only local products (Egyptian origin) are allowed to be in the tender with a CE (Conformitè Europëenne) certificate only, whereas other imported products need additional certificates.

#### Supplier reliability

To assess supply reliability, the percentage fulfilment of committed requirements in the last three years was evaluated. Suppliers fulfilling a higher percentage of committed requirements had a higher score. This criterion aims to ensure the supplier’s reliability concerning the quantities supplied and to account for any delays.

#### Previous use of the product

Medical devices that have been used before in previous tenders will receive a better score compared to others that have not been used because, according to the workshop participants, these products have been tried before, and the UPA has confidence in their quality. If the medical device has not been used in UPA tenders but was supplied to governmental or non-governmental organizations in the previous 2 years, it will receive a lower score, and the lowest score in that criterion will be received by those medical devices that have not been supplied before.

#### Instant replacement within product variety

Devices that come with instant replacement within the product variety during surgery or on shelf stock will be eligible for a better score than devices that come from less flexible suppliers.

#### Pharmacovigilance system

Each medical device supplier should have a pharmacovigilance system to be allowed in the tender. As the pharmacovigilance system becomes well established and efficient, the product receives a higher score for this criterion. The EDA will provide evidence to the UPA, for the efficiency of the supplier’s pharmacovigilance system for each medical device.

#### Refund/replacement within product variety

To ensure the replacement of unwanted or expired products, one criterion assesses the provision of refunds or the replacement of unwanted or expired products. To assess this, a stagnant report will be checked (a report that shows if a product has not been sold for the last three months). Suppliers for stagnant products receive a low score in this criterion because the product was not replaced or refunded by the manufacturer after it was stagnant for three months.

#### Weighting

“Technical characteristics of the medical device” was the highest ranked criterion; therefore, it had the largest weight of 29.4% of the decision. The “Country of origin” criterion also had a significant weight of 19.5%. These two criteria represent approximately half the weight of the decision, while the other six criteria share the other half. The least ranked criterion was “provision of refund or replacement,” with a weight of 4%. The other criteria had intermediate weights, based on their ranks. The results of the SMART and swing weighting techniques are listed in Table 2.

**Table 2:** Final criteria weights

Rank	Criteria	Weight (%)
1	Technical characteristics of the medical device	29.4
2	Country of origin	19.5
3	Use in reference countries	14.9
4	Supply reliability	11.7
5	Previous use in tenders	9.0
6	Instant replacement within product variety	6.9
7	Pharmacovigilance system	4.6
8	Provision of refund or replacement	4.0

#### MCDA tool

Following the workshop, the voting results were compiled into a user-friendly Microsoft Excel MCDA tool. Two forms of this tool were provided. A printable table that allows assessors to use the tool manually (Additional file 2: Appendix 2), and Microsoft Excel tool for automatic score calculation (Additional file 3: Appendix 3). For the printable version, the scores were adjusted based on the weights of the criteria.

To use the Microsoft Excel tool, the user should select the outcomes that match each device’s specifications from a drop-down list for the eight criteria. The tool automatically calculated the final score of the device. The tool allows for the simultaneous testing of five medical devices and calculates a score for each device to be easily compared.

To use the tool’s printable version, the user should fill the score column with a score matching the device’s performance for each criterion. Each device’s total score will be calculated through score aggregation. The same should be performed for all the assessed devices, for comparing their results.

## Discussion

Although it is challenging to compare medical devices due to their differing characteristics, materials, and exact function, MCDA tools aim to create a unified instrument for choosing between them, through the evaluable criteria's assessment.

The systematic literature review conducted showed that, MCDA tools can be used to assess several medical devices’ groups. The criteria used in MCDA tools are usually specific to the location or hospital where they are used. However, we used some general criteria, such as the “technical characteristics of the device” and “provision of training,” to develop our tool. The scoring functions of these criteria were extracted from the studies included in the systematic review. In the final tool, we attempted to include criteria with objective scoring functions, to allow for objective and flawless assessments.

Most identified MCDA tools include a price criterion. However, in Egypt, we could not add this criterion to our MCDA tool, despite its importance. In Egypt, the assessment was conducted in two phases: technical and financial. Therefore, the MCDA tool considers only the technical phase, which should be done separately before the financial phase (which includes the price). The total MCDA score achieved by the device was used for assessment based on its price.

The final weights show the experts’ extreme attentiveness towards the medical device’s quality. The first three criteria (“technical specifications”, “country of origin”, and “use in reference countries”) are related to device quality. These criteria had a collective weight of approximately 64%. When this tool is used in tenders, a low-quality device will lose a significant score for these criteria, even if its supplier provides efficient services of refund, replacement, or supply reliability. Therefore, a low-quality device would receive a final low score, which may hinder it to be the device of choice.

After the final workshop, users were provided with the MCDA tool created in Microsoft Excel based on their final ranking, weighting, scoring, and votes. From this point on, this tool can be used for medical device tenders. Medical device suppliers evaluated by this MCDA tool, should provide all the required documents for a flawless assessment. These documents include a technical characteristics sheet, quality-related certificates, manufacturing certificates, proof of all orders that have been fulfilled and the quantities sent, proof of their status towards replacing the product/s if required, and any other supporting document that may help assessors to evaluate the criteria included in the MCDA tool. The experts at the UPA will assess these documents, and if their validity is proven, the medical device will be evaluated using the MCDA tool.

## Limitations

Although the tool attempts to capture medical device specifications and details as much as possible, some aspects may still be neglected or unaccounted for. However, as per voting, the included criteria are the most important and have the most significant effect on decision-making, so specifications that were not considered are expected to have a negligible impact. In addition, similar to any other survey or collective opinion decision, the final tool may not reflect each expert’s outlook. However, averages were calculated for all voting exercises, and the tool was tried in a pilot to increase its reliability; therefore, we expect the final tool to reflect the experts’ general opinion.

## Conclusions

The MCDA tool we created could help decision makers choose between available medical devices through an objective and well-defined methodology, rather than subjective and opinion-based decisions. Determining the best option depends on the medical device’s score in the eight included criteria. Subsequently, prices should be added to the equation in the financial evaluation phase. The product that provides the lowest price per point could be considered the best value for money and should be chosen rather than competitors who might offer less value for the resources paid.

## Supplementary Information

**Additional file 1**. The detailed search term, databases, and number of hits for the systematic literature conducted.

**Additional file 2**. The final scoring table to be filled by the medical device assessor.

**Additional file 3**. The Microsoft Excel tool for comparing implantable medical devices in Egypt.

## Data Availability

All data generated or analysed during this study are included in this published article [and its supplementary information files].

## Abbreviations

CE: Conformitè Europëenne
CFG: Certificate to Foreign Government
EDA: Egyptian Drug Authority
FDA: Food and Drug Administration
MCDA: Multi criteria decision analysis
MDSAP: Medical Device Single Audit Program
SMART: Simple Multi-Attributable Rating Technique
UHI: Universal Health Insurance
UPA: The Egyptian Authority for Unified Procurement, Medical Supply, and Technology Management
