# Supplementary material for: A multicriteria decision analysis (MCDA) tool to purchase implantable medical devices in Egypt
Source: BMC Med Inform Decis Mak. 2022 Nov 9;22:289. doi: 10.1186/s12911-022-02025-y (PMC9644459; doi:10.1186/s12911-022-02025-y)
Supplement: Supplementary file 1 — Additional file 1. The detailed search term, databases, and number of hits for the systematic literature conducted. [file 12911_2022_2025_MOESM1_ESM.docx]

## **A multicriteria decision analysis (MCDA) tool to purchase implantable medical devices in Egypt**

## Additional file 1

Detailed search term, databases and number of hits for the systematic literature review conducted

| **Search engine** | **Search term** | **Hits** |
| --- | --- | --- |
| **PubMed** | (("criterion"[Title/Abstract] OR "criteria"[Title/Abstract] OR "characteristic"[Title/Abstract] OR "characteristics"[Title/Abstract] OR "attribute"[Title/Abstract] OR "attributes"[Title/Abstract]) AND  (Medical Devices [Title/Abstract] OR (Equipment and Supplies))) AND ("tendering" or "tenders" or procurement) | 133 |
| **Scopus** | TITLE-ABS-KEY ((“criterion” OR “criteria” OR “characteristic” OR “characteristics" OR "attribute" OR "attributes”) AND (medical AND devices OR (equipment AND supplies))) AND ("tendering" OR "tenders" OR procurement) | 145 |
| **Google scholar** | (“criterion" OR "criteria" OR "characteristic" OR "characteristics" OR "attribute" OR "attributes”) AND  (Apparatus and Instruments OR "Device, Medical" OR "Devices" OR "Devices, Medical" OR "Equipment" OR "Inventories" OR "Medical Device" OR "Medical Devices" OR "Supplies”) AND ( "tendering" OR "tenders" OR procurement ) | First 100  hits were screened |
