# Supplementary material for: A multicriteria decision analysis (MCDA) tool to purchase implantable medical devices in Egypt
Source: BMC Med Inform Decis Mak. 2022 Nov 9;22:289. doi: 10.1186/s12911-022-02025-y (PMC9644459; doi:10.1186/s12911-022-02025-y)
Supplement: Supplementary file 2 — Additional file 2. The final scoring table to be filled by the medical device assessor. [file 12911_2022_2025_MOESM2_ESM.docx]

## **A multicriteria decision analysis (MCDA) tool to purchase implantable medical devices in Egypt**

## Additional file 2

**Final scoring table to be filled by the medical device assessor**

| **Criterion** | **Scoring options** | **Score (choose 1 for each criterion)** | **Score**  **(To be filled)** |
| --- | --- | --- | --- |
| **Technical characteristics of the medical device** | Fulfills 100% of the technical specifications required | 29.4 |  |
|  |  |  |  |
|  | Fulfills 90%-<100% of the technical specifications required | 23.5 |  |
|  | Fulfills 80%-<90% of the technical specifications required | 17.6 |  |
|  | Fulfills 70%-<80% of the technical specifications required | 2.9 |  |
|  | Fulfills <70% of the technical specifications required | Excluded |  |
| **Country of origin** | Reference countries for both legal and actual manufacturer or local product | 19.5 |  |
|  | Reference country of the legal manufacturer or actual manufacturer | 14.6 |  |
|  | Non reference countries for both | 7.8 |  |
| **Use in reference countries** | CFG certificate from FDA | 14.9 |  |
|  | Canadian free sale certificate + ((medical device active license + MDSAP certificate) or medical device establishment license) | 11.9 |  |
|  | European CE certificate + free sale certificate from a reference country | 11.2 |  |
|  | European CE certificate only (for local products only) | 7.5 |  |
| **Supplier reliability** | Supplier fulfilled more than 90% of the committed requirements in the last 3 years | 11.7 |  |
|  | Supplier fulfilled 70% - 90% of the committed requirements in the last 3 years | 9.4 |  |
|  | Supplier fulfilled 50% - 70% of the committed requirements in the last 3 years | 7.0 |  |
|  | Did not supply previously | 5.9 |  |
|  | Supplier fulfilled < 50% of the committed requirements in the last 3 years | 1.2 |  |
| **Previous use of the product** | Listed in the UPA platform | 9.0 |  |
|  | Supplied to governmental or non-governmental organizations in the previous 2 years | 6.3 |  |
|  | Did not supply previously | 4.1 |  |
| **Instant replacement within product variety** | Supplier provides instant replacement within product variety (During surgery on shelf stock) | 6.9 |  |
|  | Supplier does not provide product replacement for different sizes/ types | 1.0 |  |
| **Pharmacovigilance system** | Supplier has an efficient pharmacovigilance system | 4.6 |  |
|  | Supplier has a moderate quality pharmacovigilance system | 3.2 |  |
|  | Supplier has a low-quality pharmacovigilance system | 0.9 |  |
|  | No pharmacovigilance system | Excluded |  |
| **Refund/Replacement within product variety** | The product was present in the stagnant report 1 time or less in the last year | 4.0 |  |
|  | The product was present in the stagnant report 2 times subsequently in the last year | 2.8 |  |
|  | The product was present in the stagnant report 3 times subsequently in the last year | 2.0 |  |
|  | The product was present in the stagnant report 4 times in the last year | 0.8 |  |
| **Total score** | | 100 |  |
